# Enhanced insulin sensitivity in successful, long-term weight loss maintainers compared with matched controls with no weight loss history

**DOI:** 10.1038/nutd.2017.31

**Published:** 2017-06-19

**Authors:** L D Clamp, D J Hume, E V Lambert, J Kroff

**Affiliations:** 1 Division of Exercise Science and Sports Medicine, Department of Human Biology, Faculty of Health Sciences, University of Cape Town, Cape Town, South Africa

## Abstract

**Background:**

Weight gain is associated with deterioration in metabolic health, whereas weight loss improves insulin sensitivity. This study assesses the impact of long-term, successfully maintained weight loss and weight-loss relapse on measures of insulin sensitivity and identifies factors that explain variability in insulin sensitivity.

**Methods:**

Women (20–45 years) were recruited into four groups: reduced-overweight/obese (RED, *n*=15); body mass index (BMI)-matched controls (stable low-weight, *n*=19), BMI⩽27 kg m^−2^; relapsed-overweight/obese subjects (REL, *n*=11); and BMI-matched controls (obese stable weight, *n*=11), BMI⩾27 kg m^−2^. A 75 g oral glucose tolerance test determined fasting and 2 h plasma glucose and insulin. Homeostatic Model Assessment (HOMA-IR) and insulin sensitivity index (ISI_(0,120)_) assessed insulin sensitivity. Anthropometric measurements, fasting resting metabolic rate (RMR) and respiratory quotient (RQ) were measured. Questionnaires and dietary intake were recorded, and physical activity was measured using accelerometers.

**Results:**

RED were more insulin sensitive, characterised by lower fasting (*P*=0.001) and 2 h insulin (*P*=0.003) levels compared with all other groups. There were no significant differences in dietary intake, sedentary, light and moderate activity, RMR or RQ in the RED compared with the other three groups. % Body weight (BW) lost (*P*<0.001), % BW regained (*P*<0.05), body fat %, light activity (*P*<0.05, only log HOMA), vigorous activity (*P*<0.05) and RQ (*P*<0.01) predicted 61.4% and 59.7% of variability in log HOMA and log ISI_(0,120)_, respectively, in multiple linear regression models.

**Conclusion:**

This study showed sustained enhanced insulin sensitivity in successful weight loss maintainers compared with BMI-matched controls with no weight loss history. Weight-loss-relapsed individuals were indistinguishable from controls. Weight loss itself was the strongest predictor of improved insulin sensitivity, whereas weight regain significantly predicted reduced insulin sensitivity. Weight-loss maintenance programs are essential to retaining metabolic benefits acquired through weight loss. Being physically active, reducing sedentary behaviour and, in particular, including small amounts of vigorous physical activity significantly predicted improved insulin sensitivity.

## Introduction

The prevalence of overweight and obesity continues to rise globally, along with associated comorbidities such as type 2 diabetes (T2DM), cardiovascular disease and certain cancers, consequently placing a heavy burden on health care provision.^1, 2^ This is evident in developed as well as developing countries where obesity is associated with the transition from rural to urban settings.^3^ Merely being overweight carries a three-fold increased risk of T2DM.^4, 5, 6^ Insulin influences energy metabolism and storage through its effect on substrate uptake and utilization along with mobilization of stored energy reserves, operating in a way that preferentially favours carbohydrate metabolism, lipid and glycogen synthesis and storage, and protein synthesis.^7^ In certain populations, long-term weight/body mass index (BMI) gain from early adulthood onwards carries an increased risk for development of T2DM even after adjusting for final BMI, suggesting that weight gain itself is associated with impaired metabolic function.^6^

Lifestyle factors have a mechanistic role in obesity and disease presentation. Obesity, combined with low levels of physical activity, is associated with intracellular lipid accumulation in the skeletal muscle and liver that impairs insulin signalling, reducing skeletal muscle glucose uptake and utilization, and weakening insulin-mediated inhibition of hepatic glucose production.^8, 9, 10, 11, 12^ Skeletal muscle alterations in response to exercise improve uptake, utilization and storage of glucose, increasing overall capacity for oxidative metabolism and reducing intramuscular lipid content, thus improving overall skeletal muscle metabolic flexibility.^13, 14, 15^ Both resistance and aerobic exercise have also repeatedly been shown to reduce intrahepatic lipid content independent of weight loss.^16, 17, 18, 19, 20^ Regular physical activity is therefore independently associated with improved insulin sensitivity both in the liver and skeletal muscle.

Weight-loss interventions using calorie restriction and/or increased physical activity have shown improvements in insulin sensitivity, with further gains achievable through a combination of both.^13, 21, 22, 23, 24, 25, 26^ Although some studies show sustained improvements in insulin sensitivity with successful weight maintenance at 12 and 18 month follow-up, other studies have shown either continued improvement or a reversal with weight regain.^27, 28, 29^ It is however unclear whether weight reduced or weight loss relapsed individuals eventually return to a level of insulin sensitivity that is in line with phenotypically similar individuals with no history of weight gain and loss or whether over the long term they are metabolically worse off as a result of this weight history. The aim of this study is therefore firstly to compare the metabolic profile of the following: (1) weight-reduced individuals; (2) overweight/obese, weight-relapsed individuals; and (3) BMI-matched controls with no history of weight loss or regain and secondly to identify any factors that might explain variations in insulin sensitivity within this sample.

## Methods

### Subject selection and screening

Recruitment advertisements were placed at local institutions and on the Sport Science Institute of South Africa website. Subjects were screened and subsequently placed into four groups. Successful weight reduction has been defined as weight loss of ⩾10%, maintained for over 12 months with weight fluctuations of 3% considered acceptable.^30, 31^ During recruitment it was stipulated that previous weight loss had to be intentional/deliberate, without the use of unregulated products, a lifestyle-related approach (diet and exercise or a combination of the two), unrelated to stress and/or anxiety and free of eating pathology. Based on these criteria, successfully reduced (RED) individuals were recruited, having previously lost ⩾15% of their body weight (BW) from a BMI⩾27 kg m^−2^ and maintained this for over 12 months with ⩽5% fluctuation from goal BW over the previous 12 months. Age-matched, stable low-weight (LSW) controls were recruited with a BMI ⩽27 kg m^−2^, but with no prior weight loss history. Weight-relapsed (REL) individuals were recruited with a BMI⩾27 kg m^−2^, having previously lost⩾15% of their BW, but subsequently regained all of this weight. Age-matched, overweight and obese stable weight (OSW) controls were then recruited with a BMI ⩾27 kg m^−2^ but no weight-loss history. Sample size was determined from a study that compared lean participants with weight-reduced and obese individuals, indicating a sample size of nine participants per group would be required to detect significant differences in fasting insulin at a significance of 0.05 and power (1−*β*) of 0.80.^32^

Participants were female, aged between 20 and 45 years. Exclusion criteria covered being pregnant or lactating, irregular menstrual cycles (defined as <7 cycles per year or cycle intervals >35days), diagnosis of a chronic medical condition and/or a condition requiring chronic medication known to affect metabolic rate (B2 agonists, β-blockers, corticosteroids and so on), finger-prick fasting blood glucose exceeding 7.0 mmol l^−1^ at screening, medication or Supplementary Appendix for weight loss, diagnosis of thyroid dysfunction or diagnosis of an eating condition. The study protocol was approved by the University of Cape Town Faculty of Health Science and Human Research Ethics Committee (HREC 214/2012). Before testing, all participants were given full information of test procedures, signed informed consent forms and were at liberty to withdraw at any time.

### Laboratory visit

Participants attended the laboratory between 6am to 9am in a fasted state. Resting metabolic rate (RMR) and respiratory quotient (RQ) were measured, along with body composition, heart rate (HR) and blood pressure. A 75 g oral glucose tolerance test was performed and thereafter participants performed an 8–10 min, single-stage submaximal fitness test and completed a number of questionnaires covering medical history, general health, reproductive history, basic socio-demographic, as well as weight history. Accelerometers were fitted and instructions given on how to wear these and were subsequently collected 7 days later. A registered dietitian guided participants through an online 24 h food recall and requested that two further 24 h food recalls were completed (covering one weekend day and two weekdays).

### Metabolic rate measurements and calculations

Subjects attended the laboratory in the morning after a 10–12 h overnight fast. RMR and RQ were measured for 20 min using the ventilated hood technique (Cosmed Quark CPET, Rome, Italy), whereas subjects rested in the supine position, in a quiet, isolated temperature-controlled (21–24 °C) room. Before each trial the metabolic cart was calibrated with a Hans Rudolph 3L syringe and analysers calibrated using normal room air (21% O_2_, 4% CO_2_ with the balance nitrogen) and standard gas mixtures (5% CO_2_, 16% O_2_ and the balance nitrogen) (BOC Special Gas, Afrox Cape Town, South Africa). RMR and total rates of fat and carbohydrate oxidation were calculated using the equations of Weir^32^ and Frayn,^33^ respectively.

### Anthropometry

Weight (BW-150, NAGATA, Tainan, Taiwan) and height were measured (3PHTROD-WM, Detecto, Missouri, USA) along with waist and hip circumference using a standard, non-elastic tape measure. Body composition was measured using Bioelectrical Impedance Analysis ((Quantum II, RJL Systems, Clinton Township, MI, USA).

### Oral glucose tolerance test blood sampling and analysis

Following fasting RMR measurements, a cannula attached to a three-way stopcock was inserted into the antecubital vein for blood sampling. A fasting blood sample (~18 ml) was drawn for the determination of fasting plasma glucose and insulin. Thereafter, the participants consumed a 75 g glucose solution and blood samples were collected at 2 h. Samples were kept on ice until centrifuged at 3,000 r.p.m. at 4 °C for 10 min and subsequently stored at −80 °C for later analysis. Plasma glucose concentrations were determined using the glucose oxidase method (Glucose Analyzer 2, Beckman Instruments, Fullerton, CA, USA). Commercial radio immunoassays were used to measure plasma insulin (Axsym Insulin Assay, Abbott Laboratories, Lake Bluff, IL, USA). Insulin sensitivity was estimated using the Homeostasic Model Assessment (HOMA-IR, using fasting glucose and insulin measures) and the insulin sensitivity index (ISI_(0,120)_, using fasting and 120 min glucose and insulin values) reflecting hepatic and peripheral insulin sensitivity, respectively.^34^ HOMA-IR and ISI_(0,120)_ were determined using the following formulae: HOMA-IR=(fasting glucose (mmol l^−1^) × fasting insulin (mU l^−1^))/22.5 and ISI_(0, 120)_=mean clearance rate/log mean serum insulin; where mean clearance rate=(75 000 mg+(0 min glucose−120 min glucose) × 0.19 × BW (kg)/120 min)/mean plasma glucose) as validated by Gutt *et al.*^35^

### Dietary intake—automated self-administered 24 h recall

Dietary intake data were recorded and subsequently analysed using the validated, automated online self-administered 24 h dietary recall (ASA24, Applied Research Programme, National Cancer Institute, Bethesda, MD, USA) based on the automated, multiple pass method.^36^ A dietitian guided participants through the first 24 h recall entry and then requested that two further days were entered. To control for variation in food consumption during the week compared with at weekends, two weekdays and one weekend day were recorded. The online ASA24 software is shown to perform well against interviewer administrated 24 h recall and in comparisons with actual energy and macronutrient intake.^37^

### Predicted maximal volume of oxygen consumption

Previously validated Ebbeling single-stage submaximal treadmill walking test was used to predict maximal volume of oxygen consumption.^38, 39^ This protocol is a low-risk test for non-athletic adults. Treadmill walking speed is determined for each participant based on their age and fitness level. Following a 4 min warm-up on a flat gradient at a speed that induced 50–70% of age-predicted maximal HR, participants continued to walk at the same speed but at 5% gradient. Steady-state HR was then determined in the final 30 s of this segment, provided HR did not fluctuate more than 5 b.p.m. in the final 2 min and maximal volume of oxygen consumption was then determined using the Ebbeling equation(38). If HR fluctuations exceeded 5 b.p.m. the participant continued on this segment for a further minute until a steady state was achieved.

### Objectively measured physical activity

ActiGraph GT3X (ActiGraph, Shalimar, FL, USA) tri-axial accelerometers were used to objectively measure physical activity. Participants wore the device on their right hip for seven consecutive days of usual activity, only removing the belt during night-time sleep, bathing, showering and swimming. A minimum of 4 days with at least 600 min per day was required for data analysis, as this is shown to provide 80% reliability.^40, 41^ Accelerometer data was downloaded, exported to Excel data tables using the ActiLife Software Version 5 (ActiLife 5, Pensacola, FL, USA) and analysed for light, moderate and vigorous activity taking place in 1 min count intervals (unbouted) using cutoff points according to Matthews and colleagues.^42, 43, 44^ These cut points were determined from studies which calibrated accelerometer counts against energy expenditure (metabolic equivalent of task), determined using a portable metabolic unit that measured the volume of oxygen consumption during a variety of tasks including lifestyle activities such as household chores. The Matthews equation stipulates that counts between 101 and 759 (inclusive, equivalent to 2–2.9 metabolic equivalent of tasks) represent light intensity physical activity, counts between 760 and 5998 (inclusive, equivalent to 3–6 metabolic equivalent of tasks) represent moderate intensity physical activity, and counts above 5999 (equivalent to over 6 metabolic equivalent of tasks) are indicative of vigorous activity.^44^

### Statistical analysis

Data were assessed for normality using histogram plots and the Shapiro–Wilks test, where *P*<0.05 indicated that data was not normally distributed. For normally distributed data mean and standard deviation were reported, a two sample *t*-test for independent groups with equal variance or a Satterthwaite’s independent sample *t*-test for unequal variance was used for two-group comparisons and a one-way analysis of variance was used for comparisons across all four groups with a Bonferroni *post-hoc* test to identify significant differences between individual groups. For non-parametric data median and interquartile ranges were reported, a Wilcoxon rank-sum test was used for two group comparisons, a Kruskal–Wallis (non-parametric) analysis of variance was used for multiple comparisons and differences between groups were assessed using a Wilcoxon rank-sum test using a Bonferroni adjustment of *P*-value for multiple comparisons (significance at 0.008, *α*=0.05/6 tests). Both HOMA-IR and ISI_0,120_ were log transformed to give a normal distribution. Pearson’s correlation coefficient and simple linear regression models were used to test for associations between variables and log HOMA-IR and log ISI_0,120_, respectively. Multiple linear regression was used to model predictors of variability in HOMA-IR and ISI_(0,120)_. The multiple linear regression models were tested for normality of residuals, linearity and homoscedasticity. Outliers were checked for influence and leverage and multicollinearity of predictors was assessed using the variance inflation factor>5. For all tests, a *P*-value<0.05 was considered statistically significant.

## Results

Participant characteristics are presented in Table 1.

Table 2 shows that OSW consumed significantly more energy than LSW (*P*<0.001) and RED (*P*=0.003), but when adjusted for BW this was not significantly different. The LSW consumed more carbohydrates and less fat than the other three groups, although only significant between the LSW and OSW. There were no significant differences in sedentary, light and moderate activity. The RED engaged in more vigorous activity compared with the other three groups (*P*=0.050), but this was only significant between the RED and OSW. In terms of overall fitness, the LSW had higher maximal oxygen consumption compared with OSW (*P*<0.05) and the RED was higher than both OSW and REL (*P*<0.05), whereas the two lean groups (RED vs LSW) were not different. There were no differences in RMR or substrate utilization between the groups.

Results of the 75 g oral glucose tolerance test (Table 3) shows that although blood glucose levels are largely comparable across all groups, the RED have significantly lower fasting and 2 h insulin levels compared with all other groups (*P*<0.005). Eight individuals recorded fasting PG⩾7.0 mmol l^−1^, a diagnostic criteria for T2DM, despite having previously had fasting blood glucose levels <7.0 mmol l^−1^ at screening. Of these, two subsequently recorded 2hr PG levels ⩾11.1 mmol l^−1^, which is a diagnostic criterion for T2DM. Removing these individuals from the analysis did not alter the results significantly.

**Figure 1:**
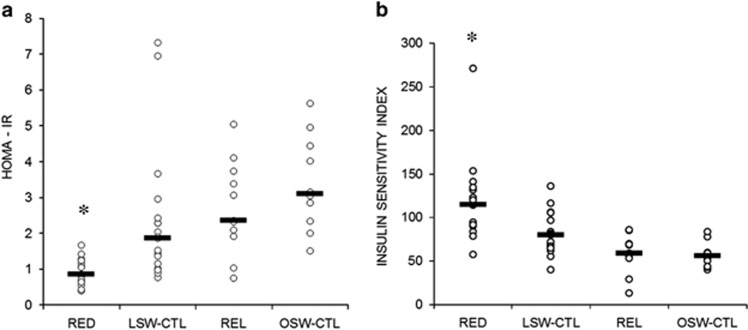
Comparison of insulin sensitivity as measured by HOMA-IR (A) and ISI_(0,120)_ (**a**) HOMA-IR (**b**) ISI_(0,120)_: *RED significantly more insulin sensitive compared with all other groups (*P*<0.001).

RED were significantly more insulin sensitive than all other groups (Figure 1). This was shown (Figure 1a) using fasting values and determining insulin sensitivity as measured by HOMA-IR (RED 0.85 (0.64–1.25), LSW 1.86 (1.01–2.43), REL 2.36 (1.91–3.73) and OSW 3.10 (2.34–4.45); *P*<0.001 for all comparisons with RED). REL were not different to either the LSW (*P*=0.138) or OSW (*P*=0.324). LSW-CTL had lower HOMA-IR values compared with OSW (*P*=0.015), whereas both the OSW and REL were not different. The same result was shown in Figure 1b using both fasting and two hour values as determined by ISI_(0,120)_ (RED 115.1 (89.8–134.7), LSW 80.0 (66.1–96.4), REL 58.7 (56.2–69.7) and OSW 55.7 (43.7–59.9); *P*<0.001 for all comparisons with RED). LSW were more insulin sensitive compared with both overweight groups (*P*<0.05), whereas the REL and OSW were not different on either measure.

The total sample was analysed to identify significant associations of variables against both log HOMA and ISI_(0,120)_ (Table 4).

The regression models (Table 5 below) were able to predict 61.4% (*P*<0.001) and 59.7% (*P*<0.001) of the variability in log HOMA-IR (Model 1) and log ISI_(0,120)_ (Model 2), respectively, in this sample. Using *β*-coefficients, the strongest predictors in Model 1 were % BW lost (*β* −0.508) followed by % BW regained (*β* 0.314) and RQ ratio (*β* 0.298), whereas for Model 2% BW lost (*β* 0.612), %BW regained (*β* −0.600) and RQ ratio (−0.231), respectively. Light activity contributed to Model 1 but was not a strong predictor in Model 2 and for Model 2 waist-to-hip ratio was a stronger predictor than % body fat. Removing the two individuals who exceeded diagnostic criteria for T2DM from the analysis did not significantly alter these results (see Supplementary Appendix).

Applying reported mean or median values for these predictors into the fitted model equation, a reduction in predicted HOMA-IR of 15% could be achieved for this sample through a 4.5% reduction in BW, a 55 min per day increase in light activity or a 5 min per day increase in vigorous activity, holding all other variables constant. For insulin sensitivity, 5 min of extra vigorous activity per day improved predicted insulin sensitivity by just 7%. However, 5% BW loss would yield predicted improvements in insulin sensitivity of 15 and 5% BW regain would reduce predicted insulin sensitivity by around 17% in this sample, holding all other variables equal.

## Discussion

This study compared metabolic, physiological and lifestyle variables across four groups of women classified exclusively according to weight status and weight loss history. The main finding from this study showed that successfully maintained, weight-reduced individuals were significantly more insulin sensitive compared with all other groups investigated. What is remarkable is that women in the RED had maintained substantial weight loss of around 15% for a lengthy period (median 30 months (12–60 months)) and yet were found to be more insulin sensitive relative to phenotypically similar, age- and BMI-matched controls with no weight-loss history. There were no significant differences in metabolic rate, substrate utilization or dietary intake that might explain the lower insulin resistance in this group, besides a modest engagement in vigorous activity. Multiple linear regression models were able to explain 61.4% of the variability in log HOMA-IR (*P*<0.001) and 59.7% in log ISI_(0,120)_ (*P*<0.001) in the total sample. In these models, previous percentage BW lost was a significant predictor of improved insulin sensitivity, independent of the current physical activity level and metabolic measures, whereas previous percentage BW regained predicted reduced insulin sensitivity. Increased fasting fat oxidation and physical activity, particularly vigorous activity, were also associated with greater insulin sensitivity (light activity was a significant predictor only in Model 1 predicting variability in log HOMA), whereas none of the dietary intake variables were found to be significant predictors.

Weight loss intervention studies have shown sustained improvements in glucose regulation at 6 month follow-up, but longer term, with weight regain these improvements were reversed.^28^ However, other groups have demonstrated that improved insulin sensitivity following weight loss was retained and even enhanced despite weight regain, and here the continued increase in adiponectin and insulin-like growth factor 1, along with no change in visceral fat, were highlighted as possible explanations for this.^29^ Our results show that individuals, who have undergone meaningful weight loss in excess of 15% of BW and subsequently maintained this well beyond 1 year and up to 5 years, are more insulin sensitive than BMI-matched controls. However, compared with RED, the REL group demonstrated lower levels of insulin sensitivity, as well as greater 2 h insulin levels in the 75 g oral glucose tolerance test, supporting existing evidence that metabolic benefits that accompany weight loss are not present in subjects who have regained weight. It is interesting to note that individuals who had always been lean and had not gone through the process of weight loss were not significantly more insulin sensitive than the REL, despite having significantly lower BMI. Undoubtedly, physiological and metabolic parameters, along with lifestyle choices, have an important role in improving metabolic health.^22, 45^ However, it is also important to recognise that individuals who have a history of substantial weight loss are evidently more insulin sensitive than those who have no weight loss history.

The RED displayed very low HOMA-IR values, with a tight clustering of individuals falling in the bottom half and even below suggested 95% reference cut points.^46^ It is therefore pertinent to consider the implications of this, particularly whether very high levels of insulin sensitivity could potentially predispose individuals to subsequent weight regain. In obese, insulin resistant populations lower relative insulin resistance is associated with increased prospective weight gain.^47, 48^ Greater weight regain following weight loss has also been shown in individuals who subsequently consumed a high glycaemic load diet.^49^ Changes in adipose tissue histology may also have a role as weight regain after sustained weight loss is accompanied by adipocyte hyperplasia.^50^ The greater number of smaller, newly reduced adipocytes are potentially more insulin sensitive, with lower rates of lipolysis and increased rates of fat storage following weight loss.^51^ This would suggest that weight cycling increases the number of adipocytes, thereby reducing the inflammatory profile of adipose tissue and improving insulin sensitivity, but also increases the efficiency and capacity for fat storage, thus raising the risk for future weight regain.^51^ This emphasises the importance of dietary recommendations and support in the weight maintenance phase following substantial weight loss in order to retain metabolic improvements.

Exercise has beneficial effects on body composition and improves insulin sensitivity through enhanced oxidative capacity and reduced intramuscular triglycerides that impair insulin signalling within the cell.^10, 12, 13^ Weight loss incorporating diet and aerobic exercise confers further beneficial effects on insulin sensitivity at 1 year follow-up that is independently associated with improved cardiovascular fitness.^52^ Both light and vigorous activity were significant predictors of insulin sensitivity as measured by HOMA-IR. There was also a strong negative correlation between sedentary time and light activity and together this demonstrates that increasing light activity at the expense of sedentary time improved fasting measures of insulin sensitivity (HOMA-IR), which is also indicative of hepatic insulin sensitivity. It is possible that with sedentary behaviour the reduced number of muscle contractions reduces skeletal muscle glucose clearance as well as lipoprotein lipase activity, resulting in reduced triglyceride clearance, thus potentially increasing ectopic fat deposition.^53^ Small amounts of vigorous activity predicted improved insulin sensitivity, which is supported by interventions showing improvements in insulin sensitivity after just 2 weeks of short duration sprint interval training.^54^ Exercise improves fasting fat oxidation which in turn improves insulin sensitivity and is confirmed in our model whereby fasting RQ significantly predicted insulin sensitivity.^55^ All groups met the ACSM Guidelines of 150 min per week of moderate to vigorous activity and this remains an important component of daily physical activity, while explicitly incorporating small amounts of vigorous activity may further improve the effect of exercise on insulin sensitivity. All participants undertook large amounts of moderate activity per day, but this did not contribute to insulin sensitivity, possibly indicating that it was lower intensity, intermittent activity rather than more structured activity over a continuous period of time. Using the regression equation generated by the model, predicted insulin sensitivity could be improved by 15% through an additional 55 min of light activity or just 5 min of vigorous activity per day.

Dietary intake variables were not found to be predictors of insulin resistance. Other studies, considering dietary glycaemic load and macronutrient composition, have also found no association with insulin sensitivity.^56, 57, 58, 59^ Other components of food intake and diet quality may be more predictive of insulin resistance than macronutrient composition *per se*.^60^ Obtaining accurate dietary intake information is inherently problematic, with day-to-day variability making it difficult to determine habitual dietary intake from three 24-h recalls.^61^ There is also some evidence of inter- and intra-individual variability in glycaemic response to the same food thereby potentially eliciting a variable insulin response.^62, 63^

One of the strengths of this study was the ability to identify individuals who had undergone substantial weight loss and either successfully maintained this weight loss for periods in excess of a year or regained all of the weight previously lost. This enabled an investigation into the longer term effects of weight loss and regain in comparison to individuals with no weight loss history. Actual measurements of physical activity also avoided issues around over or under reporting. However, limitations of the study included it being cross sectional and therefore only able to point to associations rather than cause and effect. Our research question also related to identifying differences in the metabolic profile of individuals with a weight loss and regain history compared with phenotypically similar controls and as such weight loss history was used to assign participants to experimental groups. Weight loss is accomplished through energy deficit, by increasing energy expenditure and/or decreasing energy intake. Data on how weight loss was achieved in this sample was not collected and so it is not possible to determine to what extent the weight loss associated improvements in insulin sensitivity were attributable to dietary restriction, increased physical activity or a combination of both. Finally, there were relatively fewer participants in the REL and OSW groups which may have underpowered the effects shown. Future studies should analyse the dietary data in more detail, perhaps using indices and their components to assess associations with dietary intake and insulin resistance. It would also be of interest to consider mechanisms that might contribute to explaining the improved insulin sensitivity that accompanies weight loss, to identify to what extent these might be associated with either dietary restriction or physical activity modalities and how this develops over the longer term during weight maintenance.

In conclusion, successfully weight-reduced individuals, maintaining reduced weight for extended periods of time, are more insulin sensitive than their BMI-matched controls with no weight-loss history, independent of dietary intake and physical activity. With weight-loss relapse, these metabolic benefits are no longer evidenced. Being physically active, engaging in light activity rather than being sedentary and in particular including small amounts of vigorous physical activity predicted improved insulin sensitivity. Weight-loss maintenance programs should therefore be emphasised in the period following substantial weight loss in order to retain these benefits. Research is needed to consider dietary strategies that can facilitate weight loss maintenance in light of the enhanced insulin sensitivity, not just in the immediate period following weight loss, but over the long term.

## Figures and Tables

**Table 1:** Participant Characteristics

	*LSW (*n*=19)*	*RED (*n*=15)*	*OSW (*n*=11)*	*REL (*n*=11)*
Age (years)	28a,^b^ (25–37)	32c,^d^ (26–40)	32 (29–40)	34 (22–41)
BW (kg)	59.9a,^b^ (56.8–68.3)	67.1c,^d^ (61.5–74.0)	87.6 (83.4–96.0)	92.5 (79.1–103.3)
BMI (kg m^−2^)	22.7±2.3a,^b^	24.1±2.3c,^d^	35.0±4.1	34.1±6.2
Body fat (%)	29.1±5.2a,^b^	29.6±4.3c,^d^	45.4±4.1	43.4±6.6
Fat mass (kg)	18.4±5.2a,^b^	20.4±5.3c,^d^	41.6±10.0	42.0±13.5
Fat-free mass (kg)	44.0±3.8a,^b^	47.6±4.8	49.1±5.3	52.8±5.7
Waist (cm)	68.4±4.8a,^b^	69.4±5.4c,^d^	91.8±9.0	90.7±9.6
WHR	0.69a, b (0.68–0.71)	0.66c,^d^ (0.64–0.69)	0.78 0.74–0.83)	0.75 (0.73–0.81)
				
*Weight history*				
Weight loss (%BW lost)	−2.8 (0.0–4.5)	−16.1^e^^c^ (14.4–22.0)	−1.6 (0.0–3.4)	−19.1^b^^f^ (17.3–29.5)
Weight regain (%BW regain)	0.0 (0.0–1.5)	0.0 (0.0–2.8)	0.0 (0.0–2.7)	21.0^d^^b^^f^ (15.4–26.7)
Months at current weight (months)	24.0^a^^b^	30.0^c^^d^	9.0	6.0
	12.0–60.0	12.0–60.0	6.0–12.0	3.0–30.0

Abbreviations: BMI, body mass index; BW, body weight; LSW, stable low-weight; OSW, obese stable weight; RED, reduced; REL, relapsed; WHR, waist-to-hip ratio.

Significant differences (*P*<0.05).

a LSW and OSW.

b LSW and REL.

c RED and OSW.

d RED and REL.

e LSW and RED.

f OSW and REL.

**Table 2:** Dietary intake, physical activity and metabolic measurements

	*LSW (*n*=19)*	*RED (*n*=15)*	*OSW (*n*=11)*	*REL (*n*=11)*
*Dietary intake*				
Energy (kcal per day)	1660^a^	1547^b^	2176	1572
	1297–1891	1384–2060	1955–2790	1502–2298
Energy (kcal kg^−1^)	25.±4.5	25.5±7.1	25.6±5.6	20.96±8.4
Fat (%TE)	32.1±6.9^a^	36.9±6.8	40.3±7.0	35.4±8.8
CHO (% TE)	53.4±10.0^a^	44.8±10.3	42.8±8.4	44.8±9.4
Protein (% TE)	14.0	20.2	17.2	18.8
	12.5–18.2	12.9–23.7	14.4–20.6	15.9–23.3
Protein (g kg^−1^)	0.9±0.26	1.15±0.23	1.11±0.33	1.01±0.34
				
*Physical activity*				
VO2max (ml O_2_ min^−1^ kg^−1^)	37.4±6.3	39.9±4.9	30.2±3.8	33.1±5.9
Sedentary (min per day)	1186	1118	1155	1172
	1098–1208	1038–1192	1104–1179	1104–1216
Light (min per day)	177	209	210	167
	158–247	172–275	173–249	162–205
Moderate (min per day)	79	111	86	93
	58–107	73–127	79–110	67–131
Vigorous (min per day)	0	4.1^b^	0	0
	0–0	0–11.3	0–5	0–0.125
				
*Metabolic measurements*				
RMR (kcal per day)	1,423±148	1,536±175	1,518±256	1,581±308
RMR per kg FFM (kcal kg^−1^ FFM per day)	32.4±3.3	32.3±2.4	31.1±5.2	29.9±4.6
RQ	0.76±0.06	0.76±0.06	0.78±0.04	0.79±0.05

Abbreviations: FFM, fat free mass; LSW, stable low-weight; OSW, obese stable weight; RED, reduced; REL, relapsed; RMR, resting metabolic rate; RQ, respiratory quotient; TE, total energy; VO2max, maximal volume of oxygen consumption.

Significant differences (*P*<0.05).

a LSW and OSW.

b RED and OSW.

**Table 3:** Results of the 75g OGTT

	*LSW (*n*=19)*	*RED (*n*=15)*	*OSW (*n*=10)*	*REL (*n*=11)*
Fasting PG (mmol l^−1^)	4.8	4.9	5.4	5.2
	4.6–5.1	4.5–5.2	4.8–6.2	4.7–7.5
2 h PG (mmol l^−1^)	6.1^a^ (5.3–7.3)	6.2^b,^^c^ (5.3–6.7)	7.9 (6.7–8.9)	7.7 (6.1–9.8)
Fasting plasma insulin (ml U^−1^ l^−1^)	7.6 (4.8–10.5)	4.5d,b,^c^ (2.9–5.2)	12.4 (9.1–16.2)	9.3 (5.6–14.9)
2 h Plasma insulin (ml U^−1^ l^−1^)	41.1^a^ (25.0–64.3)	19.7d,b,^c^ (10.9–31.1)	91.5 (52.1–140.2)	49.9 (31.2–115.7)
IFG: fasting PG⩾5.6 <7.8 mmol l^−1^ (*n* (% of category))	2 (10.5)	3 (20)	5 (50)	5 (45)
IGT: 2 h PG⩾7.8 mmol l^−1^ (*n* (% of category))	4 (21)	1 (7)	5 (50)	6 (55)

Abbreviations: IFG, impaired fasting glucose; IGT, Impaired glucose tolerance; LSW, stable low-weight; OSW, obese stable weight; PG, plasma glucose; RED, reduced; REL, relapsed.

Significant differences (*P*<0.05).

a LSW and OSW.

b RED and OSW.

c RED and REL.

d LSW and RED.

**Table 4:** Associations with log HOMA-IR and ISI_(0,120)_

	*Correlation coeff.*			*Correlation coeff.*	
	*Log HOMA*	*Log ISI*_*(0,120)*_		*Log HOMA*	*Log ISI*_*(0,120)*_
*Weight loss history*			*Dietary*		
BW lost (%)	−0.291†	0.253	Protein (g kg^−1^)	−0.276†	0.158
BW regained (%)	0.245	−0.319†	*Physical activity*		
*Body composition*			Sedentary time (min per day)	0.285†	−0.109
BMI (kg m^−2^)	0.477*	−0.436*	Light activity(min per day)	−0.302†	0.124
WC (cm)	0.481*	−0.479*	Vigorous activity(min per day)	−0.349†	0.263
HC (cm)	0.407†	−0.457‡	VO_2_max (ml O_2_ per kg min^−1^)	−0.429‡	0.431‡
WHR	0.362‡	−0.341*	*Substrate utilization*		
Fat mass (kg)	0.468*	−0.417†	Fasting RQ	0.338†	−0.311†
Body fat (%)	0.523*	−0.508*			

Abbreviations: BMI, body mass index; BW, body weight; Coeff: coefficient; HC, hip circumference; HOMA-IR, Homeostatic Model Assessment; ISI, insulin sensitivity index; RQ, respiratory quotient; VO2max, maximal volume of oxygen consumption; WC, waist circumference; WHR, waist-to-hip ratio.

Log HOMA-IR: BW regained: *P*= 0.071. Log ISI_(0,120)_: BW lost: *P*=0.063; Vigorous activity: *P*=0.065.

† *P*<0.05.

‡ *P*<0.005.

*P*<0.001.

**Table 5:** Regression Models predicting variability in log HOMA-IR and log ISI_(0,120)_

*Predictors*	*Model 1: predicting log HOMA-IR*				*Model 2: predicting log ISI*_*(0,120)*_			
	*Coeff.*	*95% CI*		P*-value*	*Coeff.*	*95% CI*		P*-value*
		*Lower*	*Upper*			*Lower*	*Upper*	
% BW lost	−0.038	−0.058	−0.018	<0.001	0.025	0.015	0.036	<0.001
% BW regained	0.029	0.002	0.056	0.037	−0.031	−0.045	−0.016	<0.001
Light activity(min per day)	−0.003	−0.006	−0.001	0.048				
Vigorous activity (min per day)	−0.033	−0.061	−0.005	0.023	0.015	0.001	0.029	0.043
% BF	0.019	−0.002	0.040	0.073				
RQ ratio	4.223	1.326	7.120	0.005	−1.823	−3.372	−0.275	0.022
WHR					−1.273	−2.814	0.268	0.103
Constant	−2.406	−4.589	−0.222	0.032	6.499	4.914	8.084	<0.001
Observations:	50				50			
*R*^2^ (adjusted *R*^2^)	0.614 (0.560)				0.597 (0.551)			
*P*-value	<0.001				<0.001			

Abbreviations: %BF, % body fat; %BW, % body weight; CI, confidence interval; Coeff., coefficient; HOMA-IR, Homeostatic Model Assessment; ISI, insulin sensitivity index; RQ, respiratory quotient; WHR, waist-to-hip ratio.
