# Supplementary material for: Enhanced insulin sensitivity in successful, long-term weight loss maintainers compared with matched controls with no weight loss history
Source: Nutr Diabetes. 2017 Jun 19;7(6):e282–. doi: 10.1038/nutd.2017.31 (PMC5519190; doi:10.1038/nutd.2017.31)
Supplement: Supplementary Appendix [file nutd201731x1.docx]

**Appendix:**

Multiple Linear Regression Models predicting log HOMA and log ISI_(0,120)_ – excluding 2 individuals who exceeded diagnostic criteria for T2DM for both fasting and 2 hour plasma glucose levels in the 75g OGTT.

|  | **Model 1: Predicting log HOMA-IR** | | | **Model 2: Predicting log ISI_(0,120)_** | | |
| --- | --- | --- | --- | --- | --- | --- |
| ***Predictors:*** | **Coeff** | **95% CI**  **Lower Upper** | **p-val** | **Coeff** | **95% CI**  **Lower Upper** | **p-val** |
| % BW lost | -0.039 | -0.059 -0.018 | <0.001 | 0.025 | 0.014 0.035 | <0.001 |
| % BW regained | 0.029 | 0.000 0.058 | 0.049 | -0.026 | -0.041 -0.012 | 0.001 |
| Light activity(min/d) | -0.003 | -0.006 0.002 | 0.050 |  |  |  |
| Vigorous activity (min/d) | -0.034 | -0.063 -0.006 | 0.020 | 0.013 | -0.001 0.026 | 0.068 |
| % BF | 0.019 | -0.002 0.040 | 0.077 |  |  |  |
| RQ ratio | 4.601 | 1.549 7.653 | 0.004 | -1.211 | 2.746 0.324 | <0.001 |
| WHR |  |  |  | -1.426 | -2.915 0.062 | 0.060 |
| Constant | -2.665 | -4.96 -0.370 | 0.024 | 6.157 | 4.624 7.690 | <0.001 |
|  |  |  |  |  |  |  |
| Observations: | 48 |  |  | 48 |  |  |
| R^2^ (Adjusted R^2^)  p-value | 0.620 (0.564)  <0.001 | |  | 0.578 (0.528)  <0.001 | |  |
